# Autoimmune diseases in patients with different PCOS phenotypes

**DOI:** 10.3389/frph.2026.1806450

**Published:** 2026-04-22

**Authors:** Ralitsa Robeva, Georgi Kirilov, Krassimir Kalinov, Atanaska Elenkova

**Affiliations:** 1 USHATE “Acad. Iv. Penchev”, Department of Endocrinology, Faculty of Medicine, Medical University-Sofia, Sofia, Bulgaria; 2 Scientific Affairs, Medical Center “Comac-Medical”, Sofia, Bulgaria

**Keywords:** comorbidity, PCOS, psoriasis, RA, systemic autoimmune diseases, thyroiditis

## Abstract

**Background:**

Polycystic ovary syndrome (PCOS) is a common heterogeneous condition characterized by reproductive disturbances and significant metabolic and other comorbidities. Nevertheless, the interrelations between autoimmune disorders and PCOS are still not clarified. Therefore, the present study aims to investigate the specter of systemic and organ-specific autoimmune disorders in a large group of PCOS patients, and to explore the possible interrelations between different PCOS phenotypes and the susceptibility to autoimmune disturbances.

**Methods:**

A total of 1,249 women with PCOS (18–44 years) were investigated retrospectively. The presence of autoimmune thyroiditis and other concomitant autoimmune diseases was recorded. The associations between autoimmune disorders, PCOS phenotypes, hyperandrogenism and obesity were explored.

**Results:**

The prevalence of autoimmune thyroiditis (AIT) in PCOS women was 20.3% similar to other European countries. Subclinical or overt hypothyroidism was observed in 13.45% of patients, while hyperthyroidism developed in 1%. The prevalence of AIT was not related to the PCOS phenotype, but it was increased in obese PCOS patients (23.6% vs. 18.6%, *p* = 0.045). A total of 29 of all PCOS women (2.3%) suffered from concomitant non-thyroid autoimmune disorders (NTAID). The most common NTAID in the PCOS cohort were psoriasis [6/1,249 (0.48%)], followed by type 1 diabetes mellitus (T1DM) [4/1,249 (0.32%)], alopecia areata [3/1,249 (0.24%)], and rheumatoid arthritis [3/1,249 (0.24%)].

**Conclusions:**

Organ-specific and systemic autoimmune disorders affect 22.6% of the PCOS women. The investigation of autoimmune markers in diabetic women with PCOS might be essential for the precise diagnosis. Further studies on associations between PCOS, skin and systemic inflammatory disorders might be of clinical importance.

## 1 Introduction

Polycystic ovary syndrome (PCOS) is a common heterogeneous condition characterized by chronic anovulation, hyperandrogenism, and specific ovarian morphology (1, 2). The etiology of PCOS remains unclear; however, various hereditary and environmental factors may induce steroid abnormalities and insulin resistance, which are critical for the manifestation of clinical symptoms (3, 4).

Beyond reproductive complaints, PCOS has been associated with significant metabolic comorbidity, including increased risk of type 2 diabetes mellitus (T2DM), hypertension, dyslipidemia, metabolic dysfunction-associated fatty liver disease, and cardiovascular disorders (5–8). On the other hand, non-metabolic comorbidity might also represent a significant health risk for PCOS patients suffering more frequently from sleep disorders, asthma, psychological dysfunction, as well as some types of cancer (9–11). However, the interrelations between autoimmune disorders and PCOS are still obscure.

Autoimmune diseases comprise over 150 rare or common conditions, affecting approximately one in eight individuals, with a pronounced female predominance (12–15). Certain autoimmune disturbances target specific cells, resulting in organ-specific diseases, such as type 1 diabetes mellitus; however, others affect multiple tissues, leading to systemic autoimmune involvement, as seen in systemic lupus erythematosus (12, 13). Although clinical presentations and immunological patterns might vary, the principal pathophysiological mechanism in autoimmune diseases is the breakdown of immunological tolerance. This alteration facilitates the survival of self-reactive lymphocytes, autoantibody production, chronic inflammation, and subsequent tissue damage (12, 13). Impaired immune tolerance may be associated with genetic susceptibility and environmental triggers, but sex hormones may also modulate the immune response (13, 15). Therefore, conditions associated with hormonal imbalance in women might influence the manifestation of autoimmune diseases and need further exploration.

Immune dysfunction and chronic inflammation are important characteristics of PCOS (16). The typical hormonal alterations in PCOS include various degrees of hyperandrogenism, fluctuating estrogen and prolactin levels, low progesterоne concentrations, and vitamin D deficiency, and most of these alterations may modulate immune system parameters (17–21). Usually, androgens have immunosuppressive effects; however, hyperandrogenism in PCOS may increase proinflammatory M1 macrophages in the ovary and modulate tumor necrosis factor-alpha activity (22–24). On the other hand, chronic hypoluteinism stimulates the immune response and the production of autoantibodies (16). Thus, an increase in autoantibodies, e.g., thyroid antibodies, antinuclear antibodies, and anti-ovarian antibodies, has been reported in PCOS patients in most, though not all, studies (16, 25–27). Additionally, autoimmune thyroiditis and type 1 diabetes mellitus (T1DM) have been found more often in PCOS than in healthy women (28). However, it is still not clear which hormonal or other factors in PCOS women contribute to the increased risk of autoimmunity.

Therefore, the present study aims to investigate the specter of systemic and organ-specific autoimmune disorders in a large group of PCOS patients, and to explore the possible interrelations between different PCOS phenotypes and the susceptibility to autoimmune disturbances.

## 2 Methods

### 2.1 Study participants and protocol

Paper and electronic medical files of all adult patients with PCOS attending a single tertiary Endocrine department over a 20-year period (2005–2024) were explored retrospectively. Their anthropometric, biochemical, hormonal, and imaging data were extracted from the hospital's records and pseudonymized.

Only women at reproductive age (18–44 years), of Caucasian origin, and with sufficient medical data to support PCOS diagnosis were included in the study (*n* = 1,249). The main complaints of the patients included hirsutism, acne, oligomenorrhea, infertility and/or metabolic disturbances. PCOS was diagnosed based on two of the three Rotterdam criteria [hyperandrogenism (HA), chronic anovulation (CA), and polycystic ovaries (PCO)] (1, 2). Biochemical HA was accepted in case of testosterone levels above the upper limit of method-specific reference ranges. Clinical HA was based on the presence of hirsutism, acne, and/or androgenic alopecia; women with a Ferriman-Galway score ≥8 were considered hirsute. Chronic anovulation was diagnosed based on irregular menstrual cycle or low progesterone levels in the luteal phase of the menstrual cycle in women with regular menstruation. PCO was established through ultrasound diagnostics; anti-mullerian hormone levels were not taken into consideration due to missing data for this parameter in a large proportion of study participants. Phenotyping of the patients was based on the clinical symptoms, laboratory, and ultrasound characteristics before the PCOS treatment. Women with other diseases that could lead to PCOS symptoms were excluded as suggested per guidelines, e.g., congenital adrenal hyperplasia, androgen-secreting tumors, Cushing's syndrome, acromegaly, prolactinoma, and thyroid dysfunction (1). However, patients with the same disorders were not excluded from analysis if PCOS diagnosis was established before their development or PCOS complaints persisted after their successful treatment.

PCOS phenotypes were identified based on Rotterdam criteria as phenotype A and B (HA + CA ± PCO) (“classic” PCOS, *n* = 936), phenotype C (HA + PCO) (“ovulatory” PCOS, *n* = 134), and phenotype D (CA + PCO) (“normoandrogenic” PCOS, *n* = 144) (1, 29). In 35 women, there were not sufficient data to identify the precise phenotype.

The data on anthropometric parameters were collected from medical records. Body mass index was calculated according to the formula: *weight* (*kg*)/*height* (*m*)^2^. A total of 377 women were obese (BMI ≥ 30 kg/m^2^), 840 were normal- or overweight (BMI < 30 kg/m^2^), and in 32 women, BMI was not recorded in the medical files. The use of oral contraceptives in the last 3 months and smoking status were also recorded.

The study was conducted in accordance with the Declaration of Helsinki and approved by the Institutional Ethics Committee of USHATE “Acad. Iv. Penchev” (protocol code 3/21.02.2019). Patient consent was waived by the Institutional Ethics Committee due to the retrospective character of the study and the use of pseudonymized data.

### 2.2 Autoimmune comorbidities

The data about current and past medical diagnoses, as well as clinical symptoms and medications, were extracted from the medical records of the Endocrine Department. The prevalence of autoimmune thyroid and non-thyroid disorders was studied.

The diagnosis of autoimmune thyroiditis was accepted if the patients were with already established diagnosis in the medical files or in case of at least two of the following three criteria: typical ultrasound alterations in the thyroid gland, positive immunological tests, and thyroid dysfunction (hyperthyroidism or hypothyroidism) (30). The prevalence of diagnosed and histologically proved thyroid malignancies was also recorded.

Other autoimmune diseases were considered if recorded in the medical files as preexisting or diagnosed during hospitalizations. Non-endocrine diagnoses had been made based on specific guidelines by relevant specialists. The inclusion of autoimmune diseases was based on the ICD-10 coding. The main autoimmune diseases of interest were E10 type 1 diabetes mellitus, E27.1 primary adrenal insufficiency (Addison's disease), D51.0 pernicious anemia, D59.1 autoimmune hemolytic anemia, D68.6 antiphospholipid syndrome, D69.0 Henoch Schönlein purpura, D86 sarcoidosis, G35 multiple sclerosis, G61.0 Guillain-Barre syndrome, G70.0 myastenia gravis, K50 Crohn's disease, K51 ulcerative colitis, K73 autoimmune hepatitis, K74.3 primary biliary cirrhosis, K90.0 celiac disease, L10 pemphigus, L40 psoriasis, L63 alopecia areata, L63.0 alopecia totalis, L80 vilitigo, L93 lupus erythematosus, M05-M06 rheumatoid arthritis, M08 juvenile arthritis, M32 systemic lupus erythematosus, M33 dermatopolymyositis, M34 systemic sclerosis, M35.0 Sjogrens syndrome, M35.2 Behcet's disease, M35.3 polymyalgia rheumatica, M45.0 ankylosing spondylitis, as in other studies (28).

The prevalence of different comorbidities was investigated in women with different PCOS phenotypes, obesity (BMI≥30), and the presence of hirsutism or biochemical hyperandrogenemia [increased testosterone levels at the first visit at the department (testosterone levels available in 1,155 patients)].

### 2.3 Statistical methods

#### 2.3.1 Data description

Categorical parameters were described by absolute and relative (percentage) frequencies. Continuous parameters were described by arithmetic means and standard deviations (SD), as well as median. The distribution of the continuous parameters was checked for normality using the Shapiro–Wilk test.

#### 2.3.2 Methods for testing the *post hoc* hypotheses

The Mann–Whitney U test was used to compare two independent groups. Due to the heterogeneity and non-normality of the data distributions, non-parametric ANOVA (Kruskal–Wallis) was used to compare more than two groups.

#### 2.3.3 Method used for data modeling

Binary logistic regression was used to model the relationship between the output (hypothyroidism/autoimmune thyroid disease) as the dependent variable and the main parameters, after adjustment for age. The probability of the presence of hypothyroidism/ autoimmune thyroid disease has been modeled. The odds ratios and forest plots are shown, as well 95% CIs. For decision-making, a significance level of 5% was used. Logistic regression was used because it did not rely on distributional assumptions and was considered an appropriate approach for data exploration. MedCalc® Statistical Software version 23.3.7 (MedCalc Software Ltd, Ostend, Belgium; https://www.medcalc.org; 2025) and IBM SPSS Statistics for Windows (Version 26.0) were used for calculations.

## 3 Results

### 3.1 Thyroid diseases

The prevalence of autoimmune thyroiditis (AIT) in PCOS women was 20.3% (253/1,249). Subclinical or overt hypothyroidism was observed in 168 women (13.45%), with 96 (57.1%) of them being on levothyroxine treatment. A total of 12 patients had autoimmune hyperthyroidism (1.00%). Three patients were diagnosed and treated for papillary thyroid carcinoma (0.24%), while two were born with congenital left thyroid lobe aplasia (0.16%). The prevalence of autoimmune thyroiditis did not differ between women with distinct PCOS phenotypes (“classic” phenotypes AB—20.3%, “ovulatory” phenotype C—14.2%, “non-androgenic” phenotype D—24.3%, *p* = 0.104). All hyperthyroid patients had “classic” PCOS phenotypes, though differences were not statistically significant. The prevalence of hypothyroidism did not differ between women with different phenotypes (*p* > 0.05). AIT and hypothyroidism were observed more often among obese PCOS patients (Table 1). The presence of AIT, hyper- and hypothyroidism was not associated with current OC use or smoking (*p* > 0.05 for all). The prevalence of AIT was not associated with the prevalence of increased testosterone levels among the women (*p* > 0.05), but was significantly less common in patients with hirsutism (18.4% vs. 23.9%, *p* = 0.025).

**Table 1:** Differences in disease prevalence between obese and non-obese women with polycystic ovarian syndrome (PCOS). Data are presented as mean ± standard deviation [median] or as number (percentage); *p*—differences between obese and non-obese groups calculated by Fisher's exact test. OC-oral contraceptives.

Characteristics	Non-obese	Obese PCOS	*p*
	PCOS patients	patients	
	(*n* = 840)	(*n* = 377)	
Age (years)	24.9 ± 5.2 (24.0)	26.0 ± 5.7 (25.0)	0.002
BMI (kg/m^2^)	22.9 ± 3.6 (22.5)	36.2 ± 5.3 (34.9)	<0.001
Current OC use	99 (11.8%)	29 (7.7%)	0.033
Smoking-current or former	205 (24.4%)	144 (38.2%)	<0.001
Autoimmune thyroiditis	156 (18.6%)	89 (23.6%)	0.045
Hypothyroidism	99 (11.8%)	64 (17%)	0.018
Hyperthyroidism	9 (1.1%)	3 (0.8%)	0.764
Non-thyroid autoimmune diseases	22 (2.6%)	6 (1.6%)	0.308

**Figure 1:**
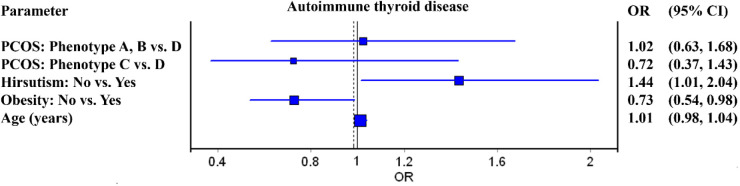
Forest plot for independent factors associated with the autoimmune thyroid disease in women with polycystic ovarian syndrome (PCOS).

**Figure 2:**
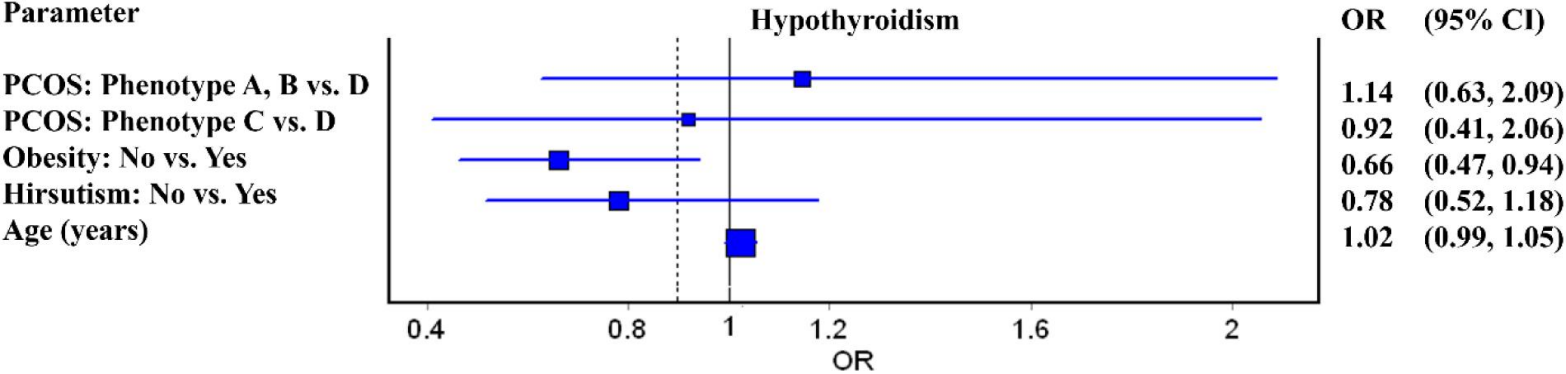
Forest plot for independent factors associated with hypothyroidism in women with polycystic ovarian syndrome (PCOS).

Logistic regression model adjusted for age showed that normal-weight patients have 27% lower risk of AIT presence compared to obese women [OR 0.73 (0.54–0.98), *p* = 0.039], while non-hirsute women were at increased risk of AIT compared to patients with hirsutism [OR 1.44 (1.01–2.04), *p* = 0.042]. Conversely, different PCOS types were not associated with AIT (Figure 1). Additionally, patients with obesity had 34% higher risk of being hypothyroid compared to normal-weight individuals, while presence of hirsutism and PCOS type were not related to hormonal disturbances (Figure 2).

### 3.2 Non-thyroid autoimmune disorders

A total of 29 of all PCOS women (2.3%) suffered from concomitant non-thyroid autoimmune (NTAID) disorders (Table 2). The most common NTAID in the PCOS women was psoriasis [6/1,249 (0.48%)], followed by type 1 diabetes mellitus (T1DM) [4/1,249 (0.32%)], alopecia areata [3/1,249 (0.24%)], and rheumatoid arthritis [3/1,249 (0.24%)], while inflammatory bowel diseases or Addison's disease were not observed. The NTAID prevalence was not increased in patients with autoimmune thyroid disease but tended to be higher in hypothyroid patients (4.2% vs. 2.0%, *p* = 0.097). The NTAID prevalence did not differ between PCOS phenotypes. Patients with NTAID were more often former or current smokers compared to non-smokers (3.9% vs. 1.7%, *p* = 0.022).

**Table 2:** Distribution of non-thyroid autoimmune diseases in PCOS patients.

Non-thyroid autoimmune diseases	29	100.0%
Antiphospholipid syndrome	1	3.4%
Alopecia areata	3	10.3%
Alopecia totalis	1	3.4%
Coeliac disease	1	3.4%
Diabetes mellitus type 1	4	13.8%
Multiple sclerosis	2	6.9%
Myasthenia gravis	2	6.9%
Psoriasis	6	20.7%
Rheumatoid arthritis	3	10.3%
Henoch-Schoenlein purpura	2	6.9%
Systemic lupus erythematosus	2	6.9%
Systemic sclerosis	1	3.4%
Vitiligo	1	3.4%

## 4 Discussion

The prevalence of autoimmune thyroiditis in the investigated PCOS women was 20.3% and did not differ from the prevalence described in a previous small case-control study of the same population (20%) (31). The prevalence was also similar to that described by other European countries using similar diagnostic criteria (20.5% in Germany and 27% in Italy) (32, 33). Recent meta-analyses have shown a two to three times increased frequency of autoimmune thyroiditis in patients with PCOS compared to controls, suggesting the need for TSH and thyroid antibodies evaluation even in patients without specific complaints (34, 35). The prevalence of clinical and subclinical hypothyroidism in the investigated PCOS group was 13.45%, while hyperthyroidism was found in 1% of women. Similar results were reported in other European and Asian studies (33, 36), whereas a recent Dutch study found much lower percentages (5.1% and 0.5%, respectively) (37). The prevalence of autoimmune thyroiditis and hypothyroidism did not differ between distinct PCOS phenotypes in our study. Similarly, Benelli et al. did not find significant associations between thyroid dysfunction and PCOS phenotypes, though high-titer anti-thyroglobulin antibodies were more frequent in women with “classic” phenotypes (38). Additionally, van der Ham et al. did not find a difference in TSH levels in women with different PCOS phenotypes (37).

In our study, obesity was a risk factor associated with autoimmune thyroiditis in PCOS patients. The interrelations between autoimmune thyroiditis, obesity, and PCOS remain highly contradictory. Some authors have found more obese women among PCOS patients with autoimmune thyroiditis compared to those without thyroid disturbances, whereas others have not reported any differences (34, 39–41). In our study, autoimmune thyroiditis prevalence among PCOS patients was not related to testosterone levels, but was lower in hirsute patients. Similarly, Ulrich et al. found higher autoimmune thyroiditis rates in PCOS patients with less severe hyperandrogenemia (39). Further studies are needed to clarify the associations among thyroid autoimmunity, hyperandrogenism, and fat mass increase in PCOS.

In our study, the prevalence of papillary thyroid carcinoma was 0.24%. Currently, studies on the associations between thyroid carcinoma and PCOS are limited. A recent Danish nationwide register study did not find an increased rate of differentiated thyroid cancer in PCOS patients compared to controls; however, there was a positive trend for thyroid neoplasm development within 10 years after PCOS diagnosis (42). Thus, further research on thyroid cancer in PCOS patients would be of clinical importance.

Non-thyroid autoimmune disorders were established in 2.3% of women in our group, and their prevalence was not associated with specific PCOS phenotypes. The most common autoimmune disorders were psoriasis, diabetes mellitus type 1, alopecia areata, and rheumatoid arthritis (RA). Currently, the associations between skin autoimmune disorders and PCOS are understudied. Only one large Taiwanese study based on National Health Insurance data has reported a doubled prevalence of psoriasis in women with PCOS compared to healthy individuals (43). On the other hand, increased prevalence of PCOS has already been observed in psoriatic patients (44). A similar Taiwanese study also reported a three-fold increase in alopecia areata prevalence in PCOS patients compared to controls (45), though data about different ethnic groups are lacking. Insulin resistance, immunologic dysfunction, and chronic inflammation have been suggested as common underlying mechanisms that could predispose to concomitant occurrence of reproductive alterations and skin autoimmune disorders (43). Recently, a large Danish study reported an increased skin disease prevalence in PCOS patients compared with controls (28); however, additional condition-specific data are needed to clarify the underlying mechanisms.

PCOS is more common in type 1 diabetes mellitus (T1DM) patients than in the common female population, with a prevalence reaching 24%–26% according to meta-analyses (46, 47). On the other hand, data focused on type 1 diabetes mellitus prevalence in PCOS are limited. Danish national-based register data have shown more than three times increased prevalence of T1DM in PCOS patients compared to controls (28). Thus, antibody evaluation in PCOS patients might be useful to ensure proper diagnosis of carbohydrate disturbances.

Studies exploring the interrelations between systemic rheumatic disorders and PCOS are scarce. According to Glintborg et al., patients with PCOS show a higher baseline prevalence of musculoskeletal systemic, and organ-specific diseases (28). A large USA study showed a non-significant increase in RA prevalence in PCOS patients compared to controls (48). Moreover, a large study based on European genome-wide association study datasets has found a causal link between PCOS and RA but not with other systemic diseases, such as systemic lupus erythematosus or polymyositis (49). Thus, more studies on patients with PCOS and RA are needed to establish the role of PCOS-driven hormonal imbalance for RA development.

The prevalence of non-thyroid autoimmune diseases in our group was not associated with obesity, in contrast to the results of North-European PCOS patients (28), but were more common in smokers. Differences between the studies might be explained by ethnic features, distinct obesity degree, and traditionally high prevalence of female smokers in the Balkan (Eastern Europe) countries.

Importantly, several limitations of the present study should be acknowledged. First, the retrospective design of the study might lead to underreporting of milder autoimmune diseases. Secondly, there was a relatively small number of patients with non-classic PCOS phenotypes, which could limit the generalizability of the results. Additionally, precise differentiation between subclinical and overt hypothyroidism was not possible in women on long-lasting L-thyroxin treatment because of the retrospective data collection. Moreover, the potential influence of long-term L-thyroxin treatment on patients' weight and degree of obesity could not be excluded. Lastly, the prevalence of non-thyroid autoimmune diseases in the group was relatively low; therefore, the role of various factors could not be reliably estimated. Nevertheless, the present study is one of the few to investigate the prevalence of autoimmune disorders in PCOS patients and to provide novel data on the Eastern European region, considering specific PCOS phenotypes.

In conclusion, our study shows that organ-specific and systemic autoimmune disorders affect at least one in five PCOS women and perhaps more, considering the possible underdiagnosis of mild autoimmune conditions. The prevalence of the most common autoimmune thyroid disease has not been related to a specific PCOS phenotype, but it has been observed more often in obese non-hirsute PCOS women. The high prevalence of type 1 diabetes mellitus in the PCOS group suggests that the investigation of autoimmune markers in dysglycemic women with PCOS might be crucial for the precise diagnosis and subsequent treatment. The possible relationship between PCOS and autoimmune skin disorders, such as psoriasis and alopecia areata, needs further evaluation. Given the retrospective design of our study, further large prospective studies are needed to confirm the observed associations and identify the specific pathophysiological mechanisms linking PCOS and autoimmune diseases.

## Data Availability

The raw data supporting the conclusions of this article will be made available by the authors, without undue reservation.
